# Association of fecal calprotectin level with eosinophilic gastrointestinal disease in Iranian pediatrics

**DOI:** 10.1186/s13223-022-00728-5

**Published:** 2022-09-30

**Authors:** Pejman Rohani, Narjes Raja Beheshti, Hosein Alimadadi, Mohammad Hassan Sohouli

**Affiliations:** 1 Pediatric Gastroenterology and Hepatology Research Center, Department of Pediatrics, Pediatrics Centre of Excellence, School of Medicine Children’s Medical Center, Tehran University of Medical Sciences, Tehran, Iran; 2 Student Research Committee, Department of Clinical Nutrition and Dietetics, Faculty of Nutrition and Food Technology, Shahid Beheshti University of Medical Sciences, Tehran, Iran

**Keywords:** Eosinophilic gastrointestinal diseases, Eosinophilic colitis, Food diet, Fecal calprotectin

## Abstract

**Introduction:**

Fecal calprotectin (FC) is a noninvasive biomarker for assessing the inflammatory status of the gastrointestinal tract. The aim of this study was to determine the association between FC levels and Eosinophilic colitis (EC) before and after treatment in pediatrics.

**Method:**

In this cross-sectional study, 330 patients with rectorrhagia and FC levels > 200 μg/g were included in the study. Patients were then subjected to colonoscopy, and if 30 or more eosinophils were observed in the pathology of at least two parts of the colon, EC was diagnosed. Of the 330 patients included in the study, 14 patients were diagnosed as EC. Treatment included seven food elimination diet (food allergens) for 3 months. After 3 months, FC levels were repeated and colonoscopy was performed.

**Results:**

The mean age of the children was 5.9 years. After the elimination diet, the number of eosinophils in all segments of colon significantly decreased (P < 0.001) and according to the pathology report, the number of eosinophils improved in 42.9% of patients. Also, the mean number of segments involved in the colon of patients was significantly decreased (P < 0.001). Mean FC levels were significantly decreased after 3 months (P < 0.001). The cut-off point of 114 μg/g of FC had sensitivity (75%), specificity (67%), positive predictive value (75%), negative predictive value (67%), accuracy (71.4%), and area under the ROC curve (0.708) acceptable in predicting EC.

**Conclusion:**

This study showed that FC levels can be elevated in patients with EC, which is easily corrected with a targeted elimination of food allergens.

## Introduction

The eosinophilic gastrointestinal diseases (EGIDs) are a rare group of diseases that can affect any part of the gastrointestinal (GI) tract [1]. EGIDs are defined as the predominance or increase of eosinophils in certain areas of the gastrointestinal tract. The prevalence of EGIDs is reported to be 1–30 per 100,000 people in the general population and 19.4 per 100,000 people in the pediatric population [2].EGIDs are classified according to the affected region of the gastrointestinal tract and include eosinophilic esophagitis (EE), eosinophilic gastroenteritis (EGE) and eosinophilic colitis (EC) [3]. EC is exceptionally rare and the clinical presentation includes abdominal pain, diarrhea (bloody or non-bloody), and/or weight loss [4].

Among EGIDs, those with mucosal inflammation tend to present with diarrhea and potentially malabsorption and bleeding, and those with muscular inflammation can present with obstruction, as well as those with serosal disease can present with eosinophilic ascites [1]. The pathogenesis of the disease is not well defined, but according to epidemiological studies and clinical forms of the disease, it is estimated that there is an allergic component. For example, it has been shown that about half of patients have an allergic disease such as asthma, food allergies, especially allergic eczema or rhinitis and in some patients high serum IgE levels [5, 6].

Currently, colonoscopy with multiple biopsies is the gold standard for assessing intestinal inflammation, but it is an invasive and expensive procedure [7]. Available blood biomarkers like erythrocyte sedimentation rate (ESR) and C-reactive protein (CRP) or serological markers such as cytoplasmic antibodies against neutrophils and anti-Saccharomyces cerevisiae antibodies are not sufficiently specific and sensitive [7, 8].

Recently, reports on the usefulness of a noninvasive test, that is, fecal calprotectin (FC) measurement in intestinal inflammation diagnosis, have been published [9, 10]. Meta-analyses of multiple studies concluded the accuracy of FC in patients with active endoscopic inflammatory bowel diseases with high sensitivity (70–100%) and specificity (44–100%) [11].

In addition to inflammatory bowel disease, elevated concentrations of FC can be seen in other pathological conditions of the GI tract such as infective colitis, microscopic colitis, eosinophilic colitis, adenomatosis, and colorectal cancer [12, 13].

Therefore, FC has emerged as a new diagnostic tool for the diagnosis and monitoring of various pathological processes in the intestinal inflammation in children, as it is a simple, rapid, sensitive, specific, inexpensive, and noninvasive marker of inflammation [13].The aim of this study was to determine the relationship between FC and EC disease before and after treatment in patients referred to Mofid Children's Hospital between 2016 and 2019.

## Methods

In this cross-sectional study, all patients (an age range of 3–10 years) with symptoms of rectorrhagia and allergies referred to gastroenterology clinics or asthma and allergies clinics of Mofid children’s hospital were included in the study between the period of November 2016 to March 2019. Symptom data were collected based on the patient’s history and with the approval of a specialist doctor. Demographic characteristics, history of allergies (included asthma, food allergies, allergic eczema or rhinitis), history of drug use, clinical manifestations of patients were recorded in specific forms for all patients. Demographic data was collected through interviews with participants by a trained expert.

Patients were referred to Boghrat Laboratory (Tehran) for FC testing. In order to collect patients’ feces, the parents of each child were asked to collect some feces in plastic containers with lids and store them in the refrigerator. Parents were told to deliver the sample to a laboratory within 7 days after stool collection and then to store it at – 20 °C for analysis. Concentrations of calprotectin in fecal samples were analyzed using EliA^®^ Calprotectin 2 (Phadia, Uppsala, Sweden) and concentrations were expressed in ug/g feces. If the FC level was above 200 µg per gram of feces, patients were referred for colonoscopy.

Patients were diagnosed with EC and included in the study and treated if there was abnormal increase of eosinophils in at least two segments of 30 eosinophils or more in the pathology report. Elimination diet is the most effective method to treat EC. However, there are different numbers of elimination diets including the elimination of 4, 6, and 7 allergenic foods, and the best effect of treatment has been reported in the elimination of more than 6 allergenic foods [14, 15]. Therefore, according to the guidelines, we considered the elimination of seven allergenic foods for the treatment of patients [14, 15]. Treatment consisted of eliminating seven allergenic foods (soy, sesame, nuts, eggs, fish, shrimp, and beef products) for 3 months [16]. After 3 months, FC was performed at Boghrat Laboratory again as well as colonoscopy were also repeated.

The response rate to the patients’ treatment was re-evaluated, and if after 3 months from the start of the patients’ treatment, 30 or less eosinophils were reported in the pathology in two intestinal segments, it was an indication of the effectiveness of the treatment.

The evaluation of the symptoms at the beginning and end of the study is based on the evaluation and clinical examinations of the patients by the specialist physician and also the questioning of the patient. If the patient is asked, the symptoms will be confirmed again by a specialist physician.

### Ethical considerations

The study was approved by the Ethical Committee of Shahid Beheshti University of Medical Sciences (IR.SBMU.MSP.REC.1398.005). Written, informed consent was obtained from the parents of all the children who participated in this study prior to their enrollment.

### Statistical analysis

Data for continuous variables are presented as mean ± standard deviations for parametric variables. Categorical variables are presented as a percentage of the total number. One-Sample Kolmogorov–Smirnov test was used to evaluate the normality of the distribution of quantitative variables. For analytical analysis, χ^2^ test, Fisher’s exact test, t-test were used. Receiver-operating characteristics plot analysis was used to evaluate optimal cutoff levels of FC.

For all statistical analyses, P value < 0.05 was considered statistically significant. All statistical analyses were performed using SPSS version 25.0.

## Results

A total of 330 patients with symptoms of rectorrhagia and FC levels above 200 μg/g were included in the study. Of these, 14 patients (4.24%) had eosinophilic colitis pathology (30 or more eosinophils in at least two colon segments) according to the pathology report.

Children with eosinophilic colitis included 7 (50%) and the mean age of children was 5.9 ± 2.2 years) range, 3–10 years). Demographic characteristics of the patients are described in Table 1.

**Table 1:** Demographic characteristics in eosinophilic colitis patients

	Total (n = 14)
Sex	
Boys	7 (50.0)
Girls	7 (50.0)
Age	5.9 ± 2.2 (3.0–10.0)
History of allergies	
Positive	9 (64.3%)
Type of allergy	
Eczema	5 (35.7)
Allergic Rhinitis	3 (21.4)
Asthma	2 (14.3)
Family history of allergies	
Positive	10 (71.4)
Total IgE serum	91.7 ± 72.4 (12.0–205.0)
Colonoscopy findings	
Nodularity	14 (100.0)

Values are expressed as count (percentage), mean ± SD

Clinical symptoms and number of eosinophils in different parts of the colon based on pathological findings in children with eosinophilic colitis before and after diet are presented in Table 2. After diet, clinical symptoms and eosinophil count in all colon segments showed a significant decrease (P < 0.001), and according to the pathology report, the number of eosinophils improved in 42.9% of patients. Also, the mean number of segments involved in the colon of patients was significantly decreased (5.9 ± 0.4 vs. 2.4 ± 2.4 and P < 0.001). In general, in 64.3% of patients, clinical manifestations (rectorrhagia, abdominal pain, diarrhea, peripheral eosinophilia, protein loss enteropathy and insufficient weight gain) were completely improved. In addition, both pathological improvement and symptomatic improvement were observed in 35.6% of patients.

**Table 2:** Clinical symptoms and eosinophil counts in eosinophilic colitis patients

	Total (n = 14)		P-value
	Before diet	After diet	
Gastrointestinal symptoms			0.001
Rectorrhagia	14 (100.0)	3 (21.4%)	
Abdominal pain	11 (78.6)	3 (21.4%)	
Diarrhea	10 (71.4)	4 (28.6%)	
Peripheral eosinophilia	3 (21.4)	0 (0%)	
Protein-losing enteropathy	1 (7.1)	0 (0%)	
Inadequate weight gain	1 (7.1)	0 (0%)	
Segments of colon (eosinophil counts in pathology)			0.001
Rectum	39.6 ± 8.1	20.1 ± 8.4	
Sigmoid	48.2 ± 12.2	24.7 ± 11.8	
Ascending	53.6 ± 17.1	29.9 ± 16.8	
Transverse	56.8 ± 22.6	30.5 ± 20.7	
Descending	64.4 ± 34.3	34.7 ± 22.4	
Cecum	72.1 ± 34.6	40.0 ± 23.3	
Average number of segments	5.9 ± 0.4	2.4 ± 2.4	0.001
Fecal calprotectin	448.0 ± 153.1	201.9 ± 186.1	0.001

**Fig. 1:**
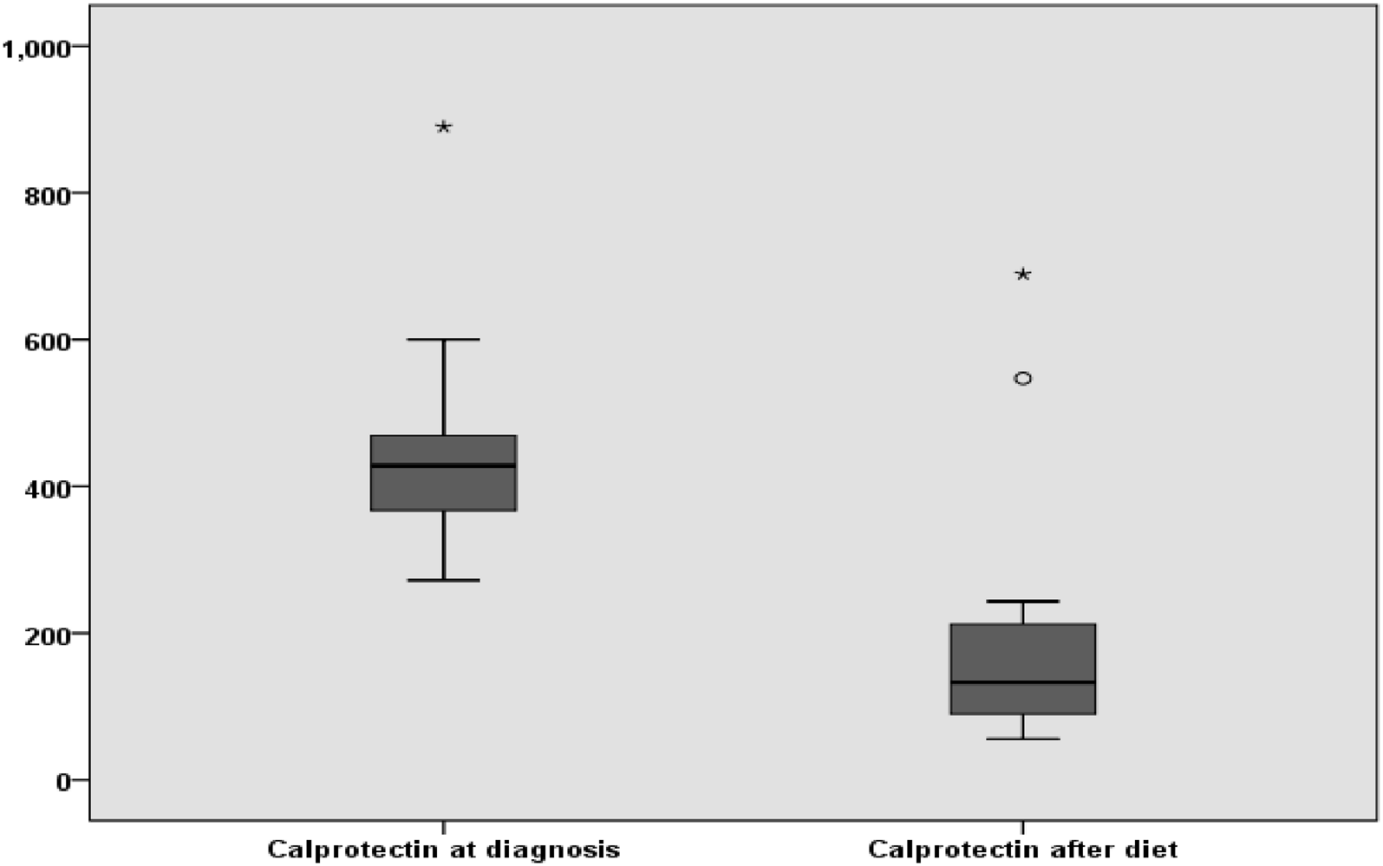
Comparison of mean fecal calprotectin in children eosinophilic colitis before and after diet

The mean FC before the intervention was 448.0 ± 153.1 (range, 890–272 μg/g) which became 201.9 ± 186.1(range, 56–689 μg /g) after 3 months of elimination diet. FC levels in patients had a significant decrease after 3 months (P < 0.001) (Fig. 1).

**Fig. 2:**
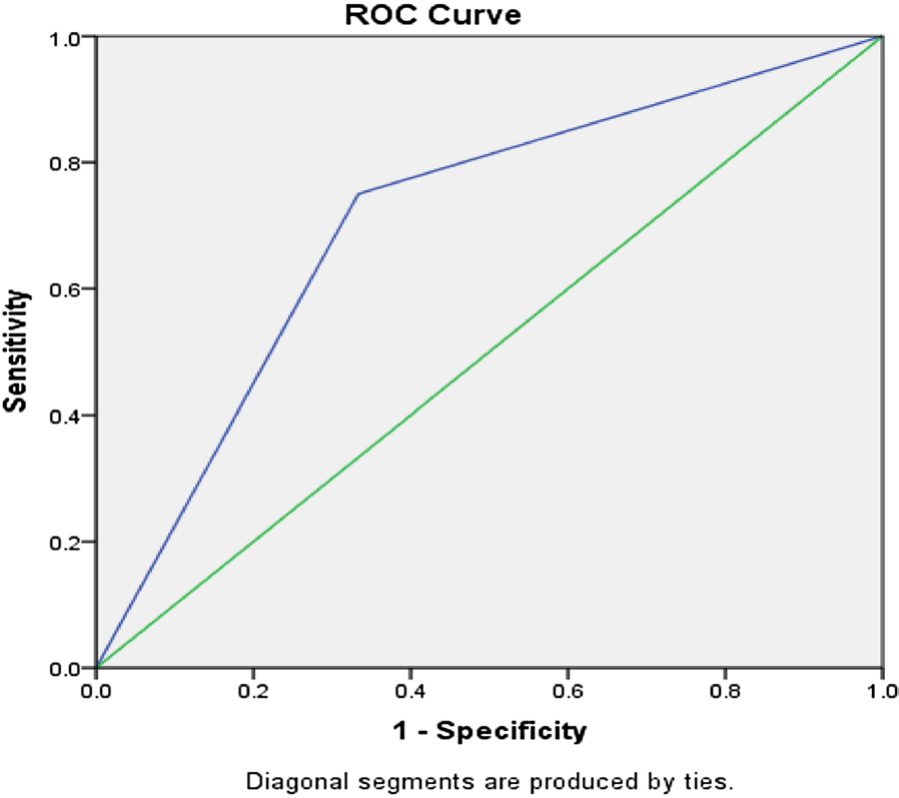
ROC curve and corresponding area under the curve of fecal calprotectin levels for predicting eosinophilic colitis, with a cut-off 114 μg/g. *ROC* receiving operating characteristic

An optimal cutoff of FC 114 μg/g in predicting eosinophilic colitis with a sensitivity of 75.0%, specificity of 66.6%, positive predictive value (PPV) of 75.0, negative predictive value (NPV) of 66.6 and accuracy of 71.4 with an area under the curve (AUC) of 0.708 (95% confidence interval [CI], 0.41–0.99) (Fig. 2).

## Discussion

The present study investigates the clinical application of FC measurement for the prediction of EC. Of 330 patients with symptoms of rectorrhagia and FC levels above 200 μg/g, 14 patients (4.24%) had eosinophilic colitis pathology (30 eosinophils or more in at least two colon segments), according to the pathology report. Our results showed that the mean FC before intervention in patients with EGIDs was 448.0 μg /g, which reached 201.9 μg/g after 3 months of elimination diet. So, the level of FC in patients decreased significantly after 3 months (P < 0.001).

The strong association of EGIDs with food allergies has prompted use of elimination or even elemental diets [4]. In one study, forty-nine children were identified with eosinophilic colitis. Elemental formula, simple elimination diet (elemental formula for infants who were on formula feeding or dietary elimination of milk, egg, peanut and wheat for those who were not on formula feeding) or combination therapy resulted in clinical improvement in 75%, 88.2% and 80% of patients, respectively [17]. In a study by Stamm et al. the use of a strict elemental diet for 3 months prior to endoscopy was not associated with a decreased frequency of eosinophilic inflammation in any tissue, which did not match the results of our study [18]. These contradictory results can be due to the type of elimination diet, the age range of the patients, and even the interpretation of laboratory data. So that the age range of the mentioned study was between 1 and 5 years, which was lower than our study (age range between 3 and 10 years). Also, interpretation of laboratory data in the Stamm et al. study was in accordance with the Boston Children's Hospital (BCH) FC normative ranges. Treatment in our study included targeted elimination of soy, sesame, nuts, eggs, fish, shrimp and beef products from patients' diets for 3 months. After diet, the number of eosinophils in all intestinal segments showed a significant decrease and according to the pathology report, 42.9% of patients had improved. Also, the mean number of segments involved in patients' intestines was significantly reduced. Clinical symptoms were completely eliminated in 64.3% of patients.

In the present study, the mean FC level of patients decreased significantly after 3 months. The next step of our study was to determine the optimal cutoff value of FC for predicting eosinophilic colitis, which revealed that a cutoff of 114 μg/g was optimal with a sensitivity of 75.0% and specificity of 66.6% at AUC of 0.708. Many authors evaluated FC concentrations in patients with suspected inflammatory process of the large intestine. Fagerberg et al. demonstrated in pediatric inflammatory bowel disease (IBD) patients that this assay is characterized by 95% sensitivity and 93% specificity, and high calprotectin concentrations show strong positive correlation with the presence of inflammatory lesions in the large intestine [19] which in some of these patients, evidence of the presence of eosinophils in the intestine was observed. Furthermore, in the study of Yoo et al. FC was useful as a noninvasive screening marker for eosinophilic gastrointestinal disorders in Korean children, which revealed that a cutoff of 73.2 μg/g was optimal for distinguishing EGIDs from Functional abdominal pain disorders (FAPD) with a sensitivity of 50.7% and a specificity of 84.6% at AUC of 0.672 [2]. In a study by Caviglia et al. FC levels in patients with macro- and/or micro-inflammation are significantly higher than those in patients without inflammation (268 μg/g vs. 49 μg/g; p = 0.0001). In nearly 40% of patients with inflammatory disease, the pathology report showed the presence of eosinophil in the digestive system in these patients. ROC curve analysis showed that an FC concentration of 150 mg/g was the best cutoff for discriminating between patients with and without inflammation. At this cutoff, FC had sensitivity = 66.7% and specificity = 90.5% [7] D’Haens et al. found the cutoff of 250 μg/g to be the most accurate predictor of the presence of ulceration on endoscopy in adult crohn's disease patients (median age of 38) [20]. According to these authors, calprotectin measurement could be a screening test preceding invasive endoscopic examinations. Differences in results and the sensitivity and specificity observed for the biomarkers examined in the studies can be due to several reasons. For example, it has been suggested that serum levels reported for FC may vary based on factors such as ethnicity, patient age, and type of biomarker kit used [21]. Therefore, the difference in the results of different studies may be due to the age difference of the participants in the study. It has also been shown that the type of kits used to measure biomarkers can produce different results [21]. So that some kits show different sensitivity to factors such as sample size and time. Therefore, it is suggested that different institutes do more research to validate the diversity between these kits before using them in clinical practice. Considering that many studies have reported the relationship between FC and gastrointestinal allergic diseases, and evidence of gastrointestinal symptoms associated with EGID has been observed in some of these patients, in another study, the association between FC as a non-invasive marker of intestinal inflammation and childhood allergic disease (atopic dermatitis) was investigated in 65 children with atopic dermatitis [22]. Among 65 children, 67/7% showed FC levels less than 50 µg/g (group 1) and 21 people (32.3%) showed higher than 50 µg/g (group 2) and on the other hand, the mean eosinophil in group 2 significantly was higher than group 1 (497.7 [239.8–1032.8]/μL vs. 281.5 [121.5–652.0]/μL, P = 0.034). Finally, the results of the study showed that the higher levels of FC observed in people with atopic dermatitis as an allergenic disease can indicate the role of FC as an inflammatory marker of the gastrointestinal tract in allergy-related diseases.

This study has several potential strengths. according to our search and information, our study was the first study that, in addition to examining FC as a useful non-invasive screening marker for EGIDs, patients with EGIDs underwent elimination diet intervention and the results of the study and the level of FC before and after the intervention were compared. Also, we tried to consider all the causes of allergies both in the patient and in the patient's family. On the other hand, we tried to consider all necessary potential include and exclude criteria and all patients entered the study with full knowledge and consent. In addition, the data related to this study was collected from a reference hospital to increase the generalizability of the results and to minimize the confounding factors regarding the patients.

One of the limitations of this study is the small sample size. Nevertheless, our study could present an optimal cutoff that can predict colitis in children with a sensitivity of 75.0% and a specificity of 66.6%. Another limitation of this study was the lack of adjustment for potential confounders such as individual characteristics such as geographical place of residence, family income, conditions during infancy or mother's pregnancy of patients and genetic factors.

## Conclusion

Our results showed high levels of FC in patients with EC at the beginning of the study, and after treating the patients with seven food elimination diet, the high level of FC was corrected. Measurement of FC in patients with colonic eosinophilia can be a safe, non-invasive and useful diagnostic marker. The cut-off point of 114 μg/g of FC has a sensitivity (75%), specificity (67%), PPV (75%), NPV (66.6%), accuracy (71.4%), and area under the ROC curve (0.708) in predicting EC disease. Overall, it seems that FC may prevent unnecessary colonoscopies, however, confirmation of this finding requires further studies with stronger designs and larger sample sizes.

## Data Availability

Data available on request due to privacy/ethical restrictions.
